# HIV-2 Vpx neutralizes host restriction factor SAMHD1 to promote viral pathogenesis

**DOI:** 10.1038/s41598-021-00415-2

**Published:** 2021-10-25

**Authors:** Ahlam Mohamed, Talal Bakir, Huda Al-Hawel, Ibtihaj Al-Sharif, Razan Bakheet, Lubna Kouser, Valarmathy Murugaiah, Maha Al-Mozaini

**Affiliations:** 1 Immunocompromised Host Research Section, Department of Infection and Immunity, King Faisal Specialist Hospital and Research Centre, PO Box 3354 (MBC-03), Riyadh, 11211 Kingdom of Saudi Arabia; 2 Department of Clinical Laboratories Sciences, College of Applied Medical Sciences, King Saud University, Riyadh, Saudi Arabia; 3 Imperial College London, London, UK; 4 Biosciences, College of Health, Medicine and Life Sciences, Brunel University London, London, UK

**Keywords:** Infectious diseases, Viral pathogenesis, Pathogenesis, Infectious diseases

## Abstract

SAMHD1, a human host factor found in myeloid cells which restricts HIV-1 replication. It depletes the dNTPs pool for viral cDNA syntheses, thus preventing the viral replication in the cells. The viral accessory protein, Vpx, exists only in SIVmac/HIV-2 particles. Vpx in SIVmac can induce proteosomal degradation of SAMHD1, which then leads to a decrease in the cytoplasmic dNTP pool. The protein–protein interaction between Vpx and SAMHD1 and its consequences are still unclear. Methods: In this study, we cloned, for the first time, Vpx gene from a HIV-2 infected patient and found up to 30% sequence variation compared to known HIV-2 strains. We then analyzed the role of SAMHD1 protein expression in transfected THP-1 and U937 cells by transfecting with the Vpx gene derived from SIVmac, HIV-2 from the NIH sample as well as HIV-2 from a Saudi patient. We found that Vpx gene expression led to reduced levels of intracellular SAMHD1. When the supernatants of the transfected cell lines were examined for secreted cytokines, chemokines and growth factors, Vpx expression seemed to be suppressive of pro-inflammatory response, and skewed the immune response towards an anti-inflammatory response. These results suggest that Vpx can act at two levels: clearance of intracellular restriction factor and suppression of cytokine storm: both aimed at long-term latency and host–pathogen stand-off, suggesting that Vpx is likely to be a potential therapeutic target.

## Introduction

Replication of HIV-1 and other retroviruses is dependent on host cell protein response. Some host cell proteins act as restriction factors to resist infection and replication. Sterile-motif and HD-domain containing protein 1 (SAMHD1), a host restriction factor, is a deoxynucleoside triphosphate triphosphohydrolase (dNTPase), which interferes with HIV-1 infection in non-cycling immune cells^1,2^. SAMHD1 is highly expressed in human myeloid-lineage cells and CD4+ T lymphocytes. Dendritic cells (DCs) and macrophages are principal immune cells required for recognizing HIV-1 at the mucosal site. Thus, these cells are less permissive to HIV-1 infection, as compared to activated CD4+ T-cells due to host restriction factors which limit the HIV-1 infection. SAMHD1 regulates intracellular dNTP pools, and thus, restricts the replication of HIV-1 in permissive cells, including non-activated CD4+ T cells. Therefore, understanding the molecular basis of such host restriction as a part of host innate immunity can offer insight into how to develop novel therapeutic drugs against HIV-1.

HIV virus can counteract SAMHD1 by using antagonistic accessory proteins such as Vpx. SAMHD1 prevents the infection of host cells by retroviruses, including HIV, via depletion of the cellular dNTP pool available for viral reverse transcription^3–5^. SAMHD1 is ubiquitously expressed in a range of human cells/tissues^6,7^. SAMHD1 is overcome by HIV-2 and related SIVs (simian immunodeficiency virus) by the different viral proteins, such as Vpx and Vpr^8,9^. Vpx links to SAMHD1 via DDB1/DCAF1-dependent E3 ubiquitin ligase for ubiquitination and proteasomal degradation. Furthermore, Vpx proteins from different SIVmac lineages target distinct regions of SAMHD1 but recruit it to the same E3 ligase^10^, as evident from the two recent crystal structures^11,12^. The anti-viral activity of SAMHD1 is negatively regulated by phosphorylation at residue T592^7,13^; however, the phosphorylation-dependent regulatory mechanism is unclear. It is possible that phosphorylation causes a reduction in the dNTPase activity of SAMHD1. Additionally, SAMHD1 also possesses exonuclease activities on ssDNA or RNA^7,13–15^, which can potentially lead to HIV restriction^16^.

It has been shown that Vpx proteins from SIVmac and HIV-2 promote HIV-1 infection in human DCs and macrophages; this occurs due to enrichment of HIV cDNA^17–20^ via enhanced and efficient reverse transcription^21^. Vpx from SIVmac or HIV-2 can bind DCAF1 in the CUL4A/DDB1 and E3 ubiquitin ligase complex^3,22–24^. HIV-1 lacks Vpx protein, but use of Vpx via lentiviral vectors has helped understand how myeloid-lineage cells become reasonably resistant to lentiviral infection/transduction^4^.

SAMHD1 is a novel Vpx-binding protein that is also expressed in differentiated human monocytic THP-1 cells^24^. SAMHD1 is expressed in HIV-1 non-permissive cell types, including THP-1 cells, primary monocytes, monocyte-derived macrophages and DCs, but not in HIV-1 permissive CD4+ T-cell^24^ Knockdown of endogenous SAMHD1 protein in non-permissive THP-1 cells and primary DCs alleviates HIV-1 restriction. By contrast, overexpression of SAMHD1 in PMA-differentiated monocytic U937 cells inhibits HIV-1 infection^3,13,24^. As described above, Vpx interacts with SAMHD1, leading to proteasomal degradation of SAMHD1 in THP-1 cells or macrophages; this can be interfered with by proteasome inhibitor^3,24^. Vpx from HIV-2 or SIVmac places SAMHD1 onto the E3 ubiquitin ligase complex, CRL4/DCAF1, to initiate its proteasomal degradation^25^. A novel DCAF1-binding motif required for Vpx-mediated degradation of SAMHD1 protein has been reported^26^.

In this study, we have cloned a Vpx gene from a HIV-2 infected patient and transfected the Vpx gene in THP-1 (SAMHD1+) and U937 (SAMHD1−) macrophage cell lines. Vpx containing plasmids originated from SIVmac (pSIVmac), Saudi patient HIV-2 (pHIV-2-Patient), and a reference HIV-2 from NIH Vpx (pHIV-2-NIH). We examined the sustenance of SAMHD1 in the macrophage cell lines, and their subsequent immune response following Vpx transfection via cytokine/chemokine/growth factor multiplex analysis.

## Results

### Vpx gene cloned from Saudi patient shows high degree of diversity compared to known strains

**Figure 1:**
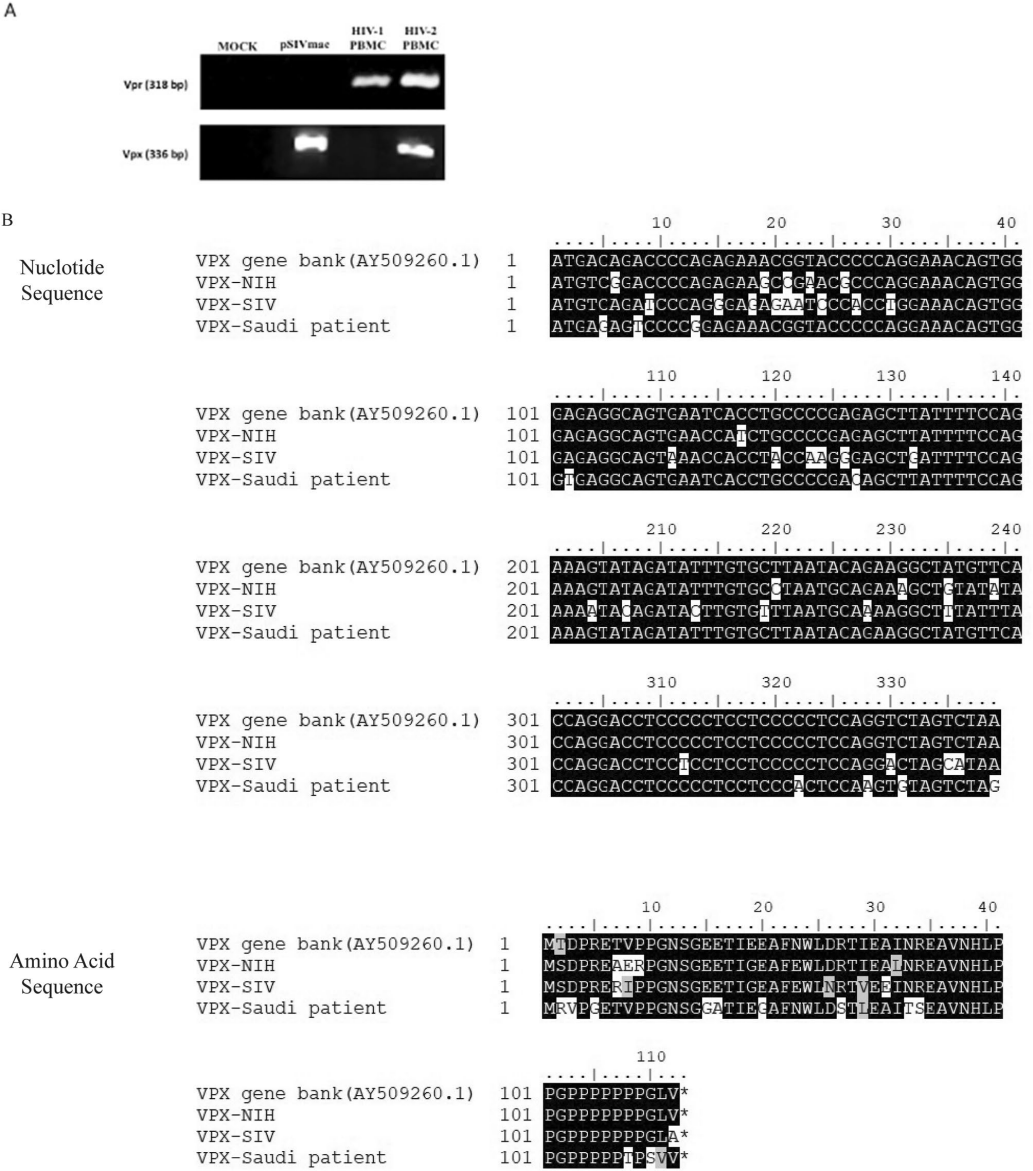
Amplification product of Vpx from patient’s sample and the Sequence analysis of different cloned Vpx genes (**A**) PCR amplicon product for Vpr and Vpx genes from HIV-1 and HIV-2 PBMCs patient samples. Vpr amplicon product can be seen present in both HIV-1 and HIV-2 samples, thus, used as control; however, Vpx gene is amplified only in HIV-2, to confirm the viral type. (**B**) The sequence alignment analysis for both nucleotide and amino acid sequence of the cloned Vpx genes from SIVmac (Rhesus HIV), HIV-2 virus stock from NIH and HIV-2 virus from one of the Saudi patients. The sequence alignment revealed that the Vpx gene derived from Saudi patient infected with HIV-2 was highly divergent.

We first cloned the Vpx gene from a Saudi patient sample using a nested PCR approach as Fig. 1A shows the nested PCR approach using terminal primers. In order to confirm the Vpx and Vpr genes at the genomic level obtained from HIV-1 and HIV-2 PBMCs patient’s samples, genomic DNA was extracted from these patients. As shown in Fig. 1A (see Supplementary Fig. S1) Vpr, 318 base-pair (bp) was detected in HIV-1 as well as HIV-2 samples. However, Vpx gene amplified only in HIV-2 and SIVmac (pSIVmac), confirming the existence of Vpx gene (336 bp) in HIV-2 infected Saudi patients. We subsequently sequenced the Vpx gene obtained from Saudi patient and carried out sequence alignment (using DNASTAR) for recombinant DNA (pSIVmac, pHIV-2-Patient and pHIV-2-NIH). It revealed that both cloned Vpx from NIH and Saudi patient had a high level of sequence homology with 92% similarities in nucleotide sequences with the human Vpx gene sequence deposited at the gene bank (accession number: AY509250.1), amino acid sequence homology of 84%, and sequence diversity of > 30% with pSIVmac (Fig. 1B).

### Vpx transfection substantially reduces SAMHD1 expression level in SAMHD1 positive THP-1 macrophage-like cells

**Figure 2:**
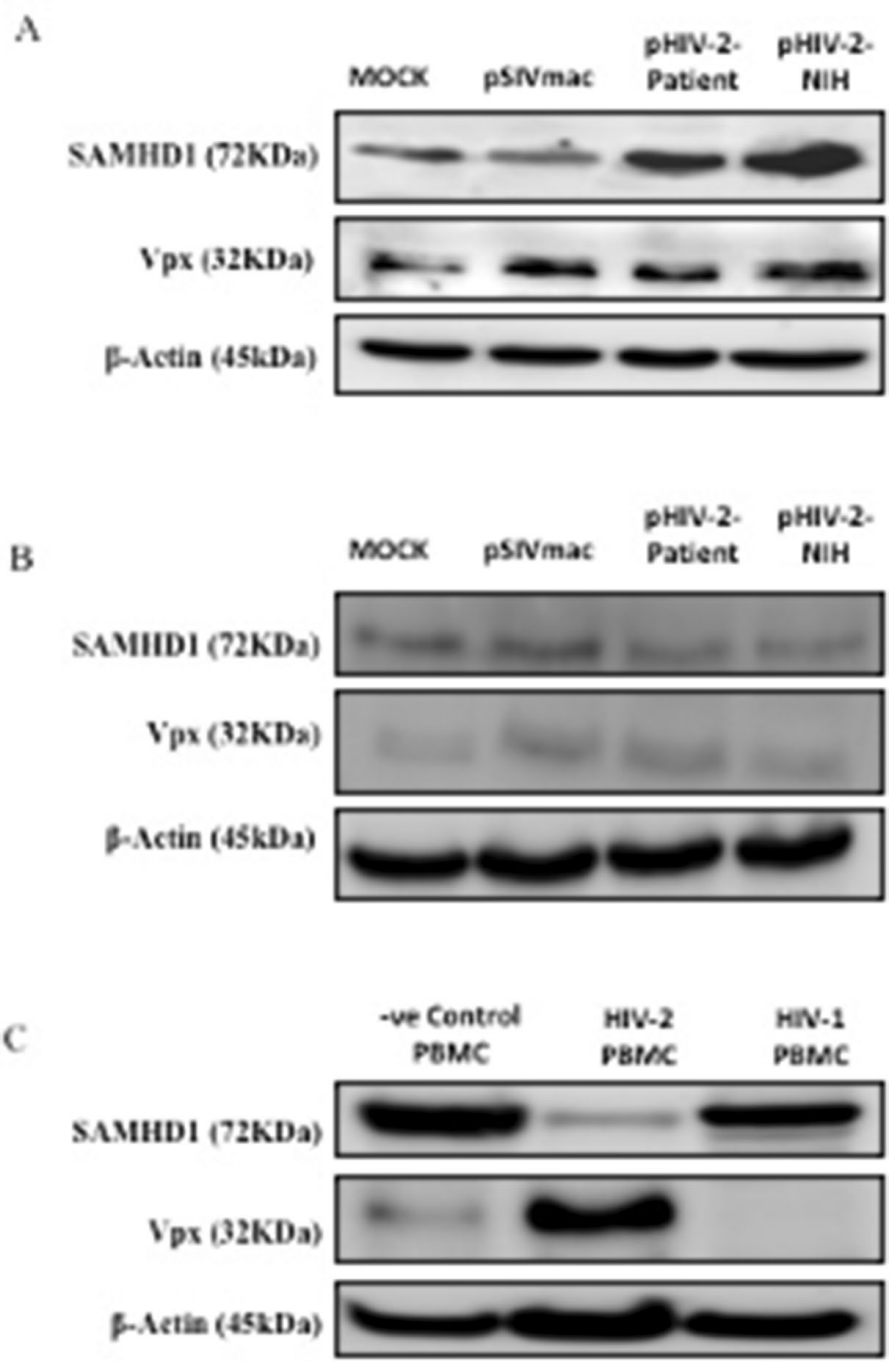
Western blot to probe SAMHD1 expression with and without Vpx. (**A**) THP-1 and (**B**) U937 cells transfected with pSIVmac-Vpx, pHIV-2-Patient, pHIV-2-NIH and mock plasmid were incubated for 24 and the western blot was probed using anti-SAMHD1 and anti-Vpx monoclonal antibodies; anti-β-actin was used as a housekeeping positive control. It is worth noting that THP-1 cells have SAMHD1 proteins while U937 cells are negative for SAMHD1 protein. (**C**) Western blot was also done for different PBMCs sample from HIV-1, HIV-2 and healthy negative control donor cells. Anti-SAMHD1, anti-Vpx and anti-Beta actin were used. As shown in Vpx was express in HIV-2 PBMCs cells protein. SAMHD1 in HIV-2 has low express compared to HIV-1 and donor cells protein. β-actin was used as housekeeping gene control.

To investigate the role of Vpx gene on SAMHD1 expression, we selected SAMHD1 negative THP-1 and SAMHD1 positive U937 cell lines and transfected Vpx plasmid originating from SIVmac, NIH strain and Saudi patient. Twenty-four hours post-transfection, total cell extracts were subjected to western blot. The proteins were probed with anti-SAMHD1 and anti-Vpx monoclonal antibodies separately. As a negative control, plasmid without Vpx insert was used (Mock). As a positive housekeeping control for the western blot, β-actin was used. The results indicated that knockdown of Vpx gene had minimal to no effect on SAMHD1 expression in THP-1 cells, while Vpx absence seems to have a strong effect on the expression level of SAMHD1 (Fig. 2A) (see supplementary Fig. S2). Transfected cells with pSIVmac showed reduced SAMHD1 expression most likely due to proteosomal degradation by Vpx. In the Saudi patient and NIH reference Vpx panel, the level of SAMHD1 expression was relatively weaker. Surprisingly, SAMHD1 was not detected in the samples originating from U937 cells (Fig. 2B) (see supplementary Fig. S3). However, these cells also expressed the transfected Vpx gene feebly while expression of β-actin was not affected (see Supplementary Fig. S5).

### SAMHD1 and Vpx interaction and expression levels in PBMCs derived from HIV-1 and HIV-2 patient PBMCS

To identify the cellular proteins associated with Vpx and SAMHD1 that may play a role in HIV replication, we examined the consequences of the expected interaction of SAMHD1 and Vpx protein extract from PBMCs derived from HIV-1 and HIV-2 infected patients. As shown in Fig. 2C (see supplementary Fig. S4), Vpx was expressed in HIV-2 PBMCs cells. On the other hand, SAMHD1 was expressed to a lesser extent in HIV-2 PBMCS cells compared to HIV-1 and donor cells. These results indicate that SAMHD1 and Vpx have differential expression patterns at the protein levels. The inverse relationship observed between the levels of Vpx (~ 33 kDa) and SAMHD1 (~ 72 kDa) appears to support our hypothesis that Vpx is neutralizing SAMHD1.

### Flow cytometry analysis confirmed the reduction of SAMHD1 expression in Vpx-transfected THP-1 cells

**Figure 3:**
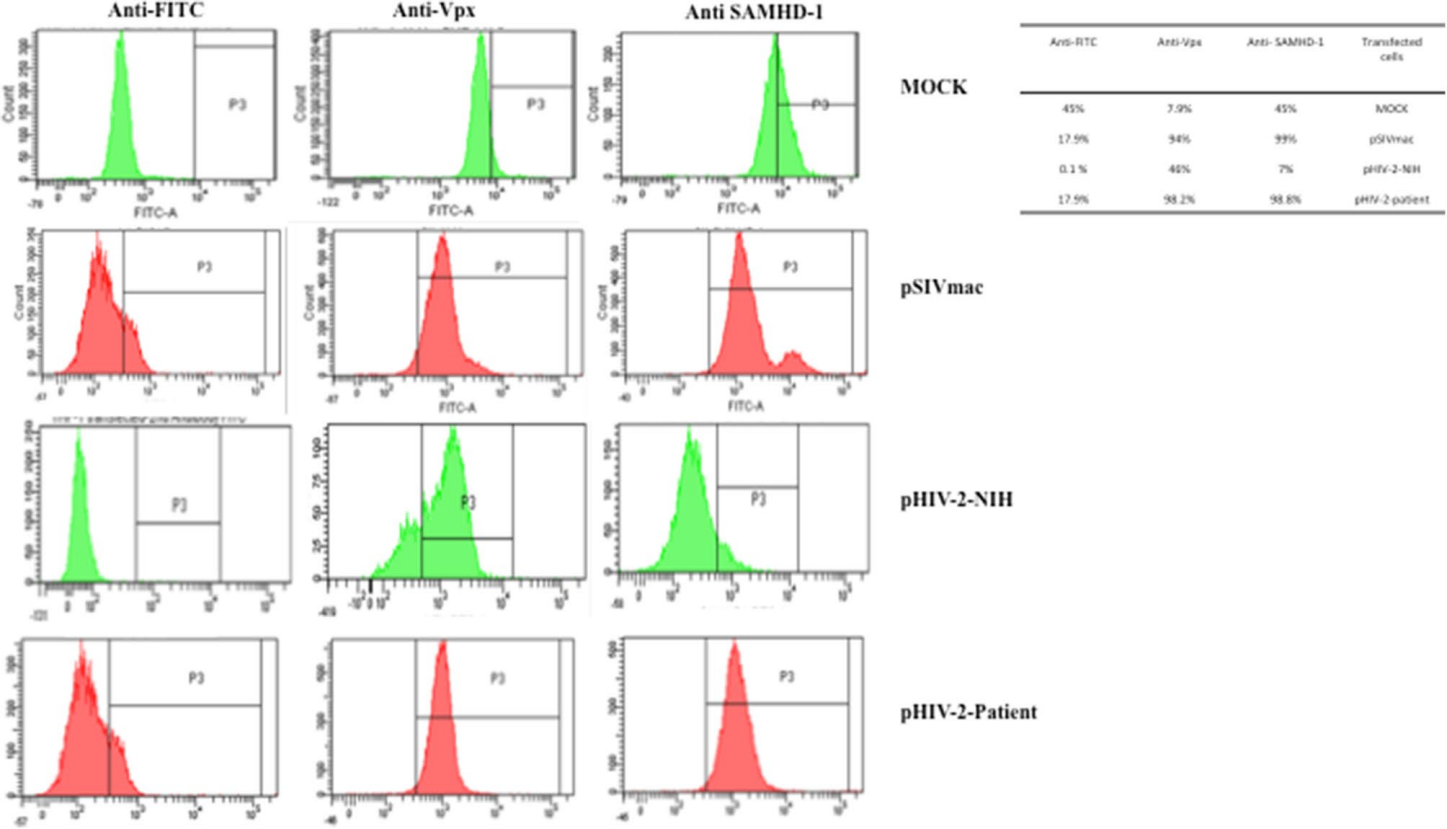
Flow cytometry for THP-1 transfected with different Vpx gene. Negative THP-1, THP-1 transfected pSIVmac-Vpx, THP-1 transfected pHIV-2-Vpx (Patient) and THP-1 transfected pHIV2-Vpx (NIH), SAMHD1 and Vpx antibodies and anti FITC secondary antibody was added.

To confirm the results obtained in Fig. 2, THP-1 cells were transfected with Vpx constructs derived from SIVmac (pSIVmac), HIV-2 Patient (pHIV-2-Patient and the HIV-2 NIH (pHIV-2-NIH) and cultured for 24 h, fixed, permeabilized, and analyzed by Flow cytometry (Fig. 3) using anti-SAMHD1 as well as anti-Vpx monoclonal antibodies in separate single-color staining.

The SIVmac-Vpx transfected cells showed clear positive staining for Vpx as well as SAMHD1. However, the most striking effects were noticeable in HIV-2 (NIH) transfectants where Vpx expression led to considerably reduced level of detection of SAMHD1 in permeabilized THP-1 cells. However, these effects were consistent in Saudi patient HIV-2 Vpx transfectants but not as dramatic as the reference NIH strain.

### Multiplex cytokine/chemokine array analysis yields clue for HIV-2 latency

**Figure 4:**
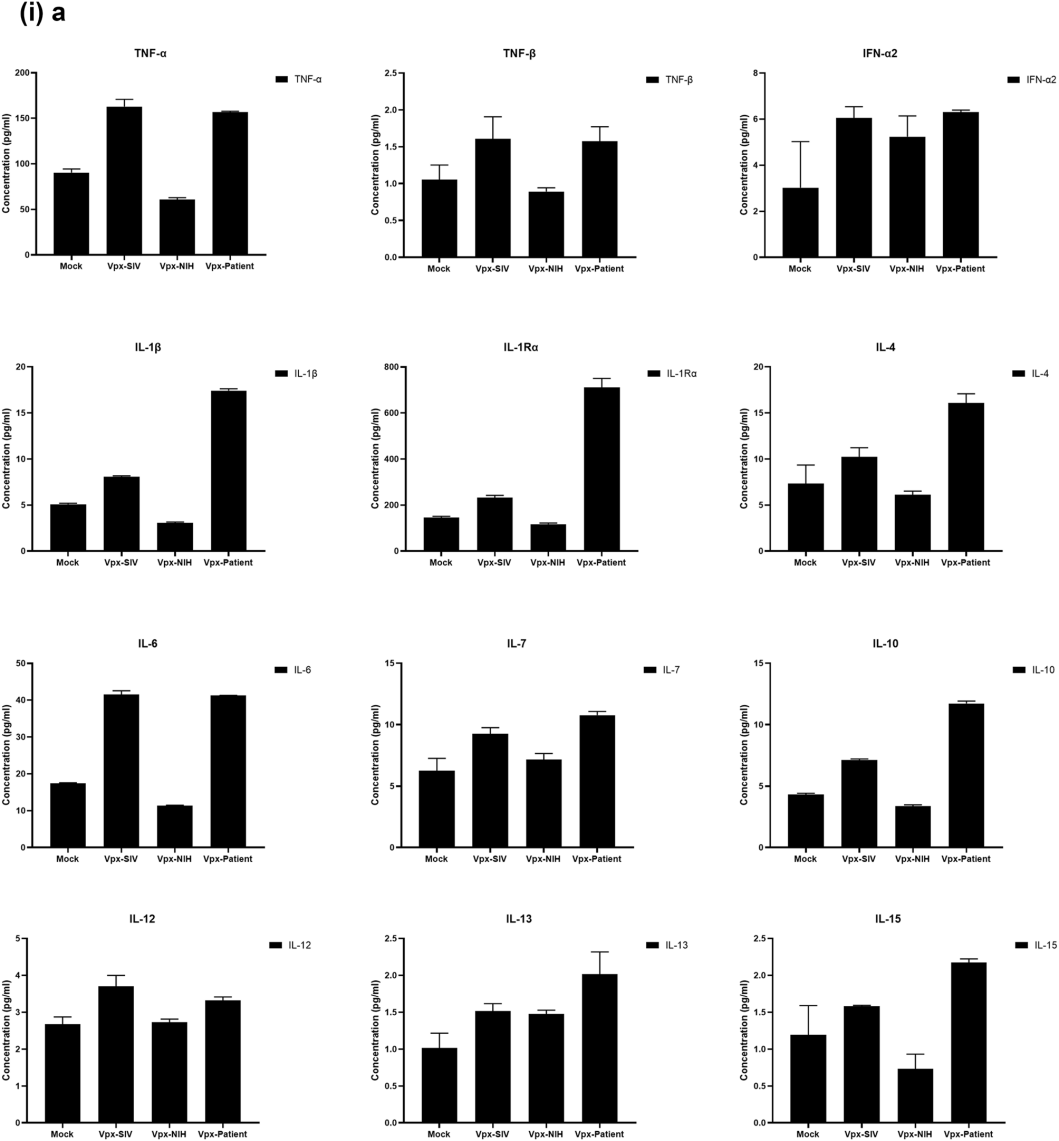

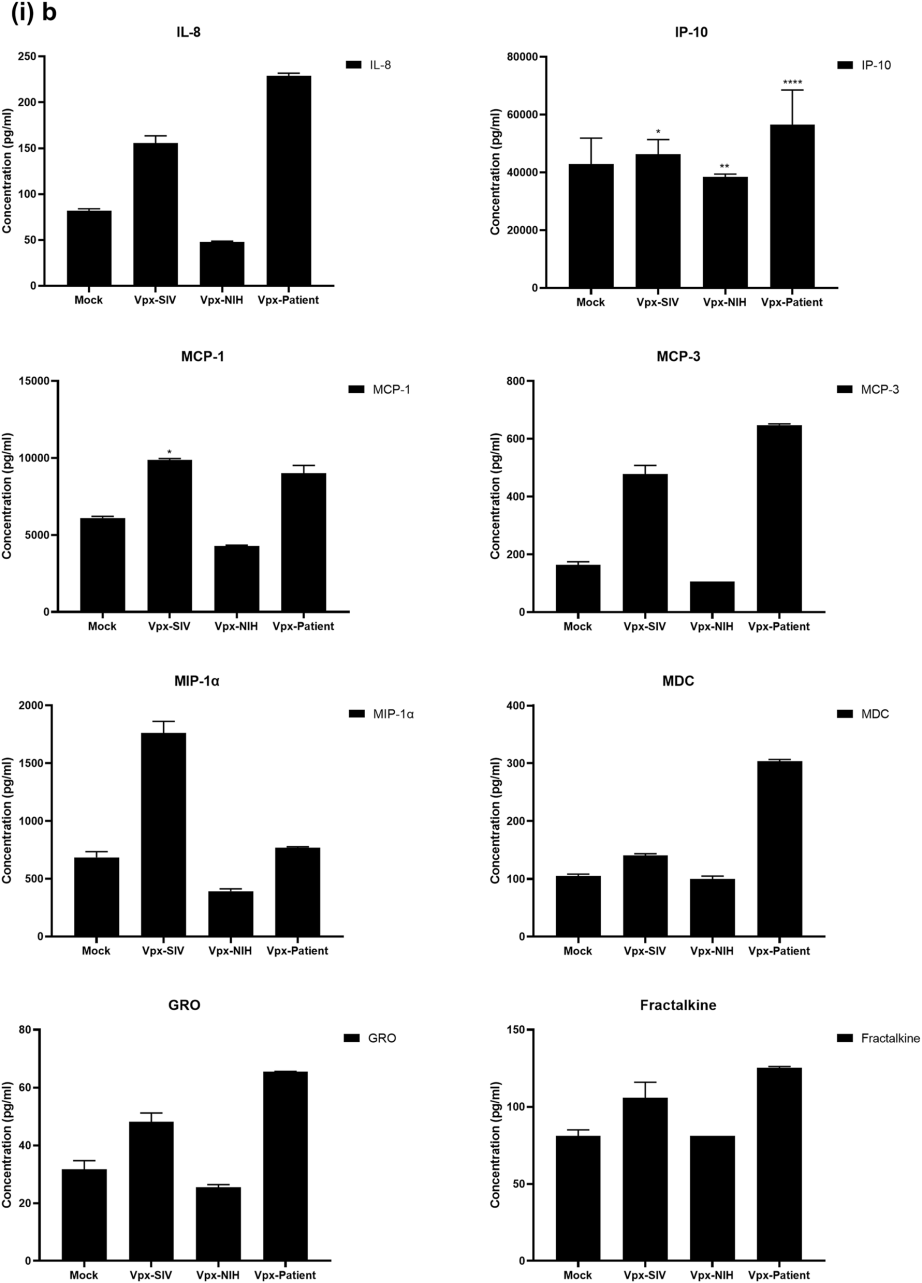

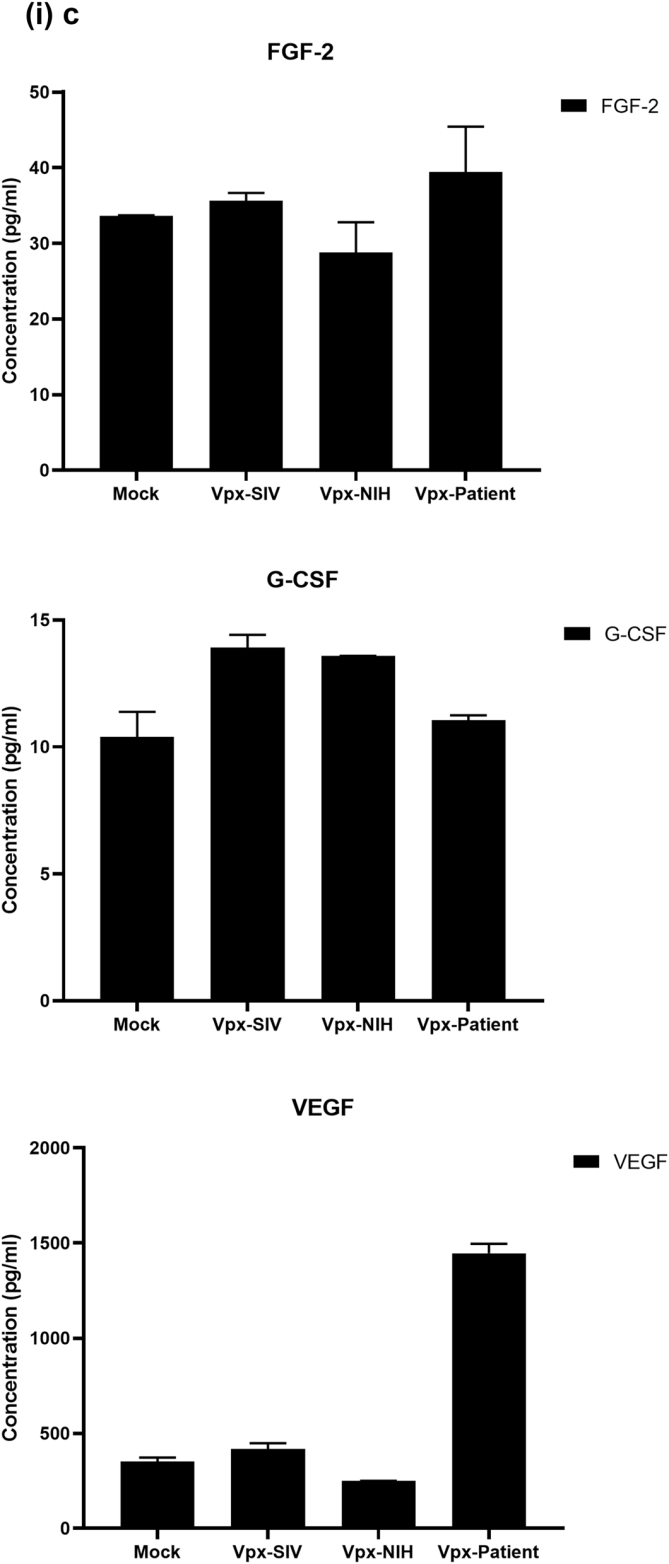

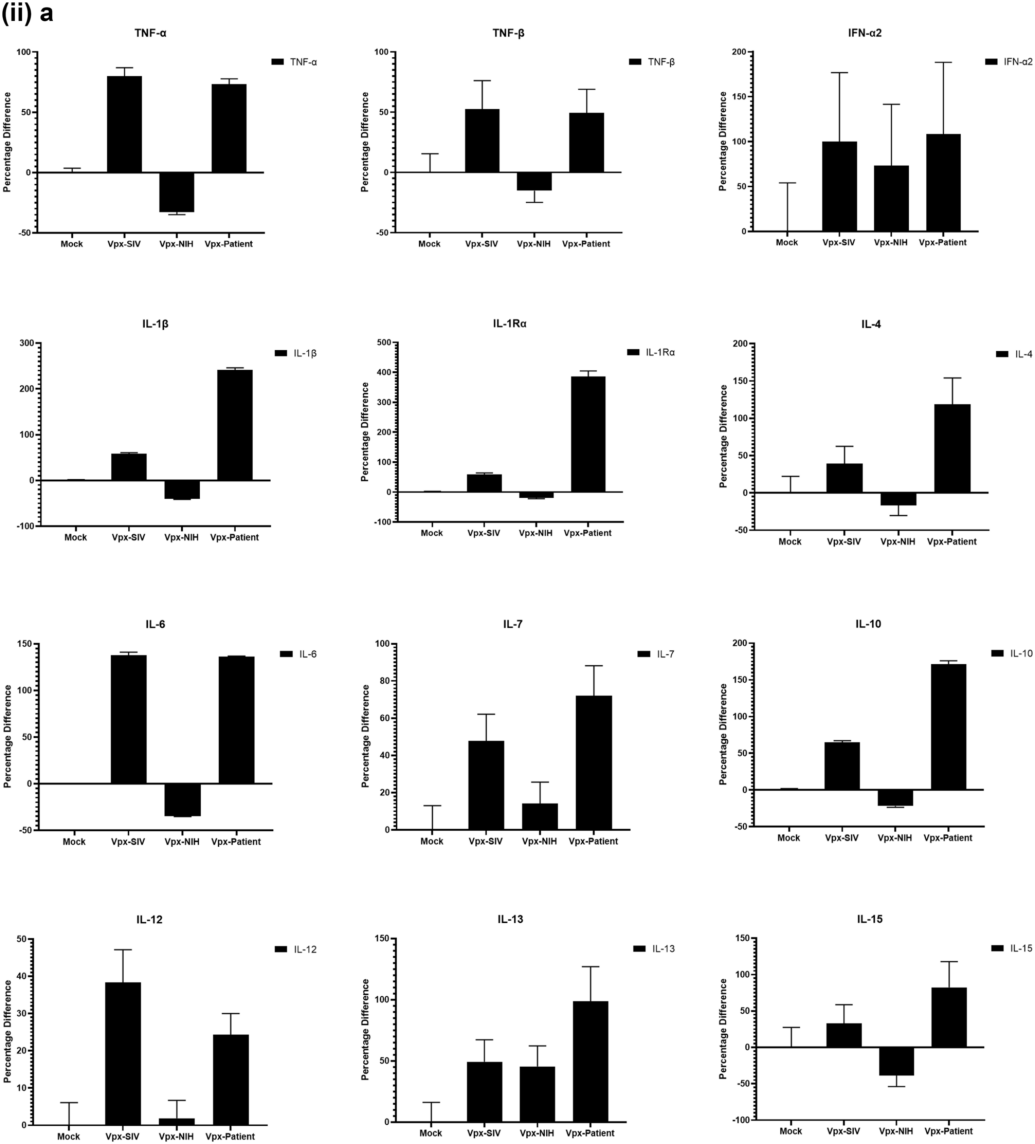

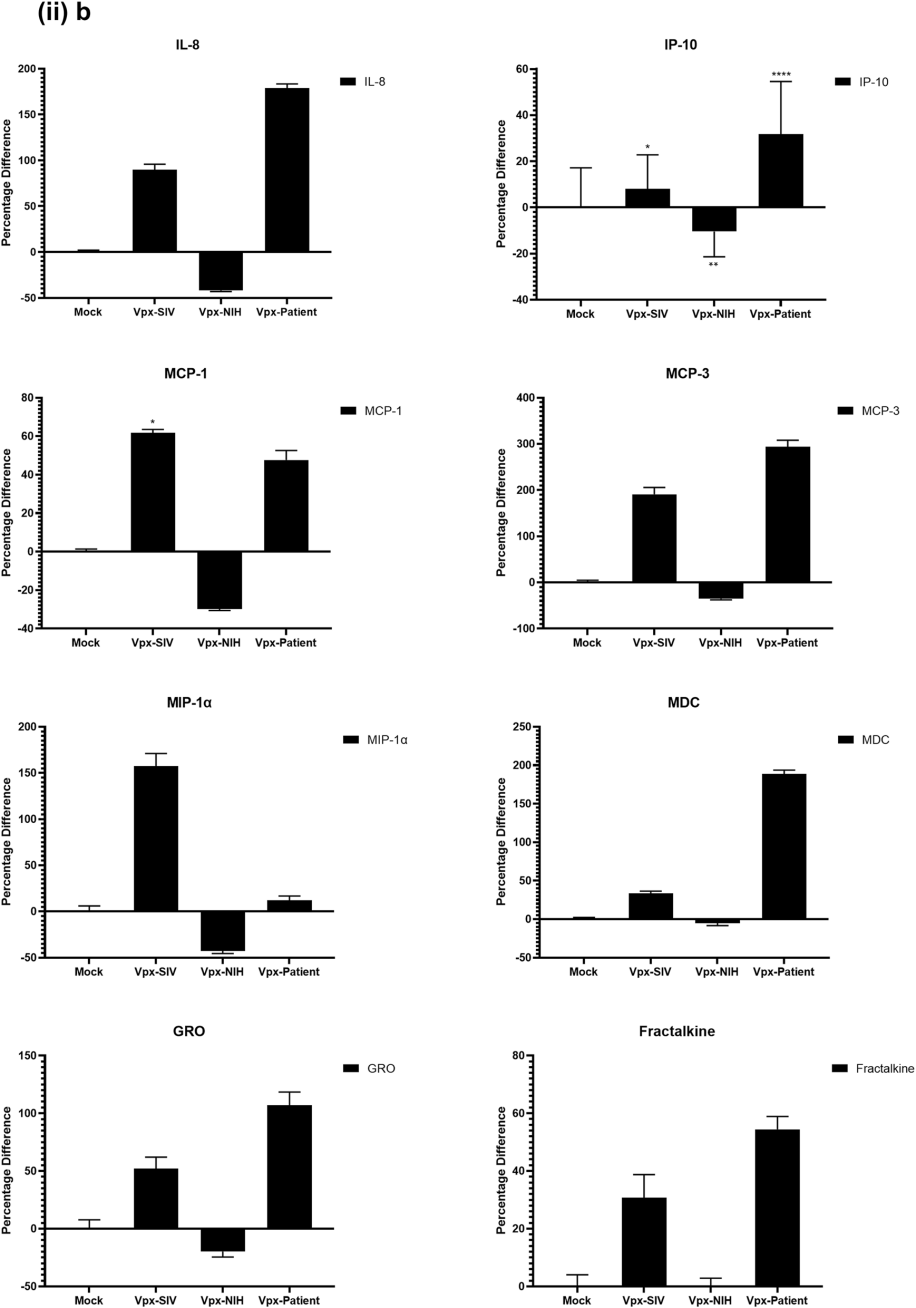

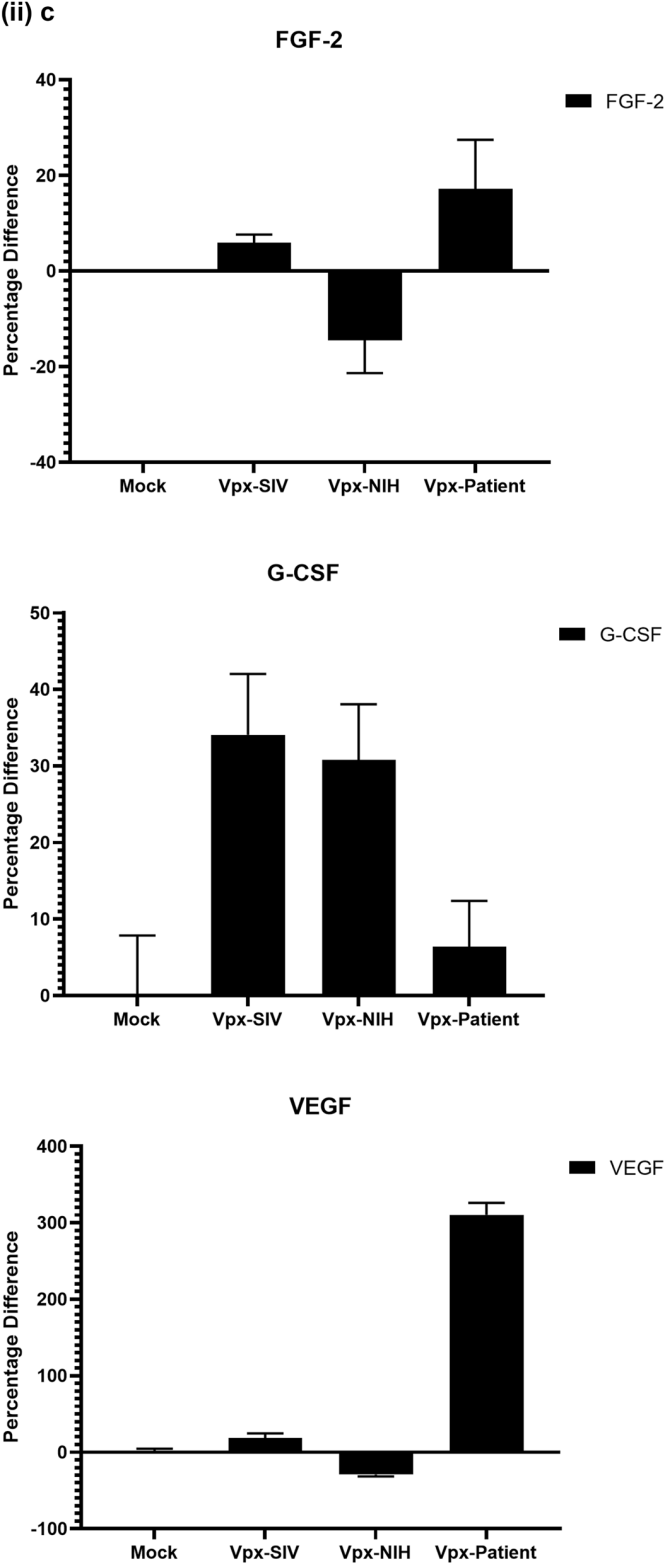
Multiplex cytokine array analysis of supernatants of THP-1 cells following transfection with pSIVmac-Vpx, pHIV-2-Vpx (NIH), pHIV-2-Vpx (Patient) and mock plasmid. Supernatants were collected after 24 and subjected to analyte analysis by Multiplex cytokine array analysis. (**i**) set represents actual values while (**ii**) represents normalised values against mock and expressed in percentage. Graph (**a**) represents the Cytokines, (**b**) represents Chemokine and (**c**) represents different levels of growth factors.

**Figure 5:**
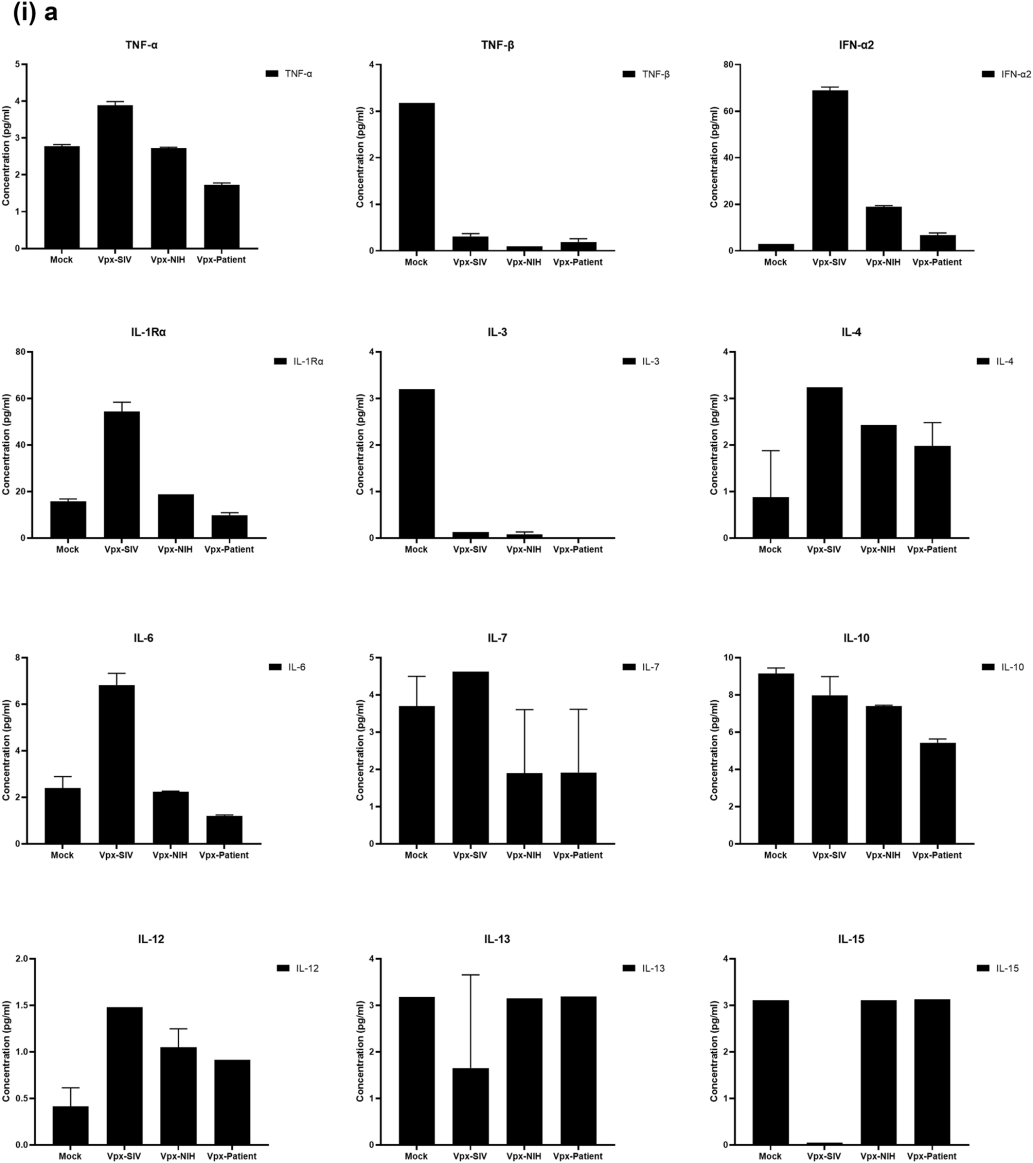

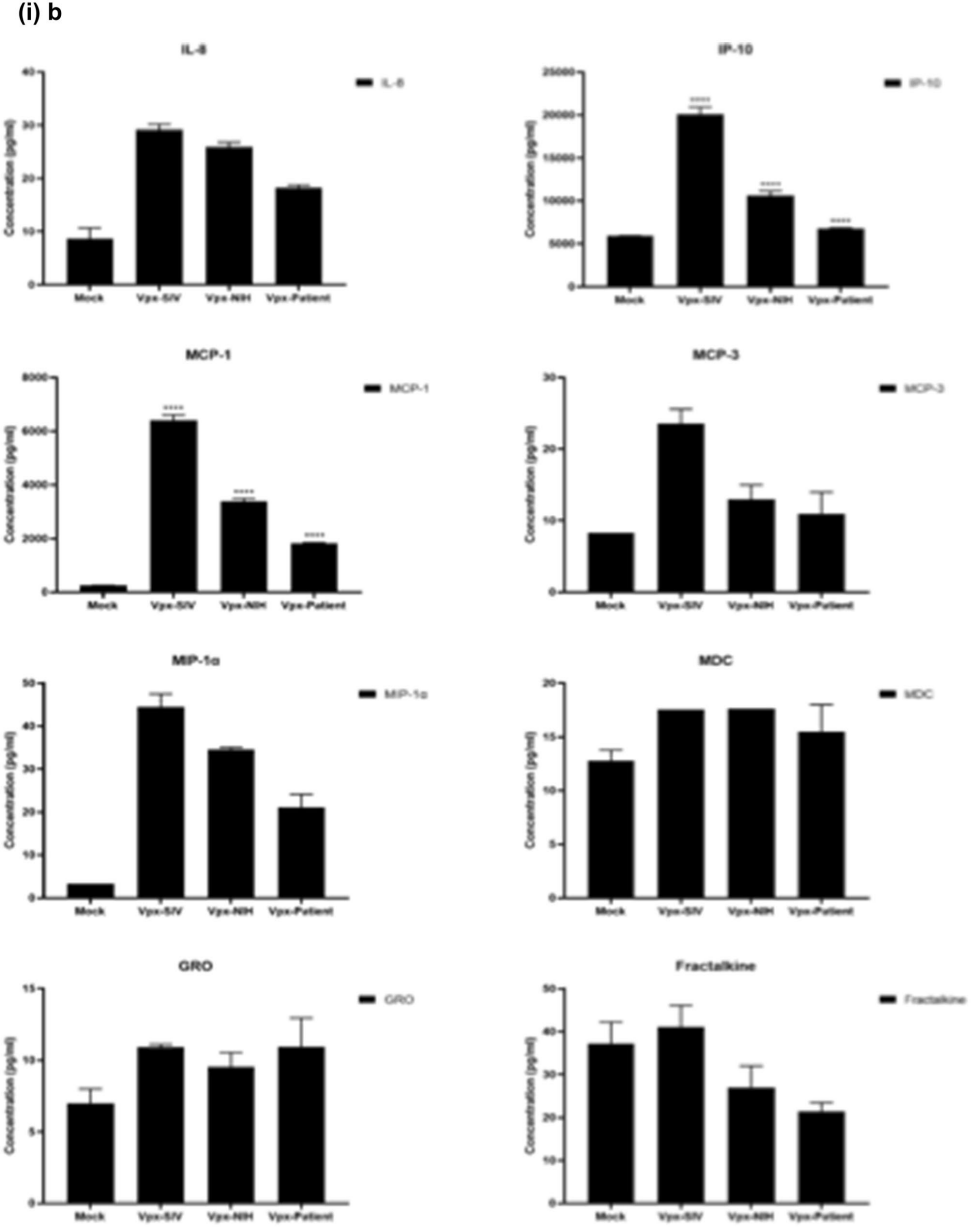

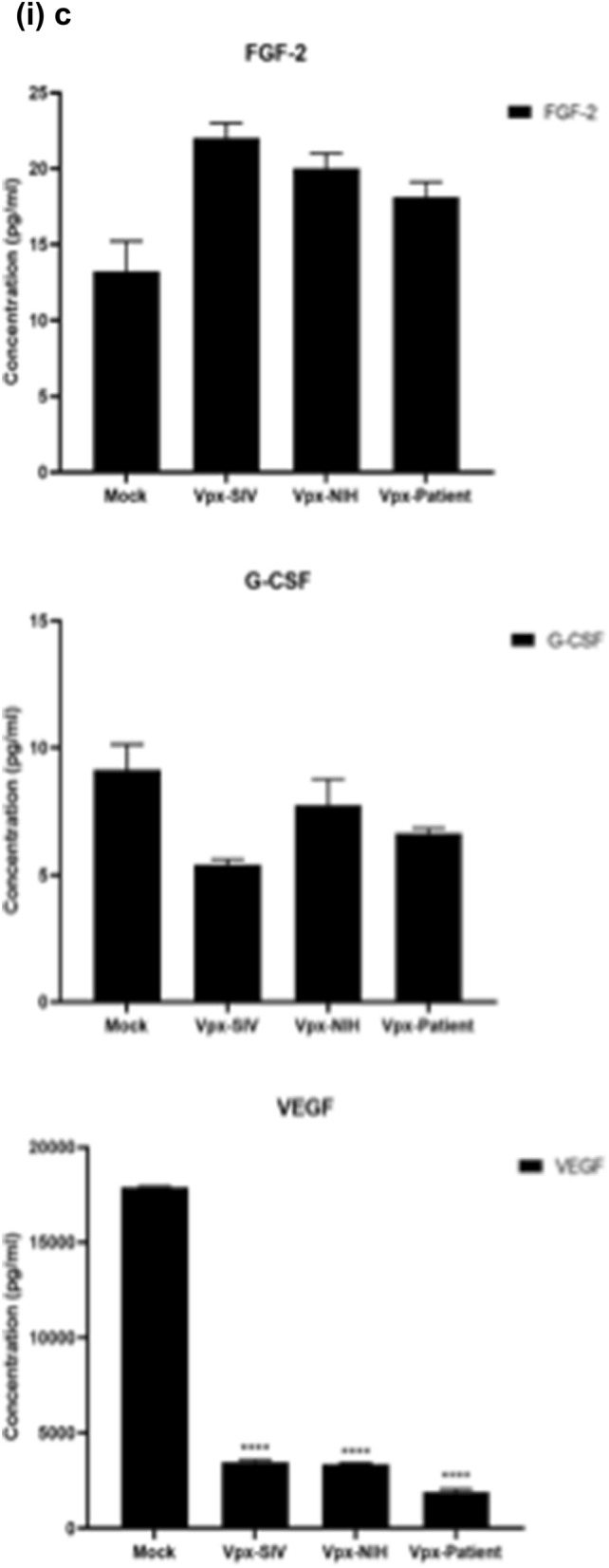

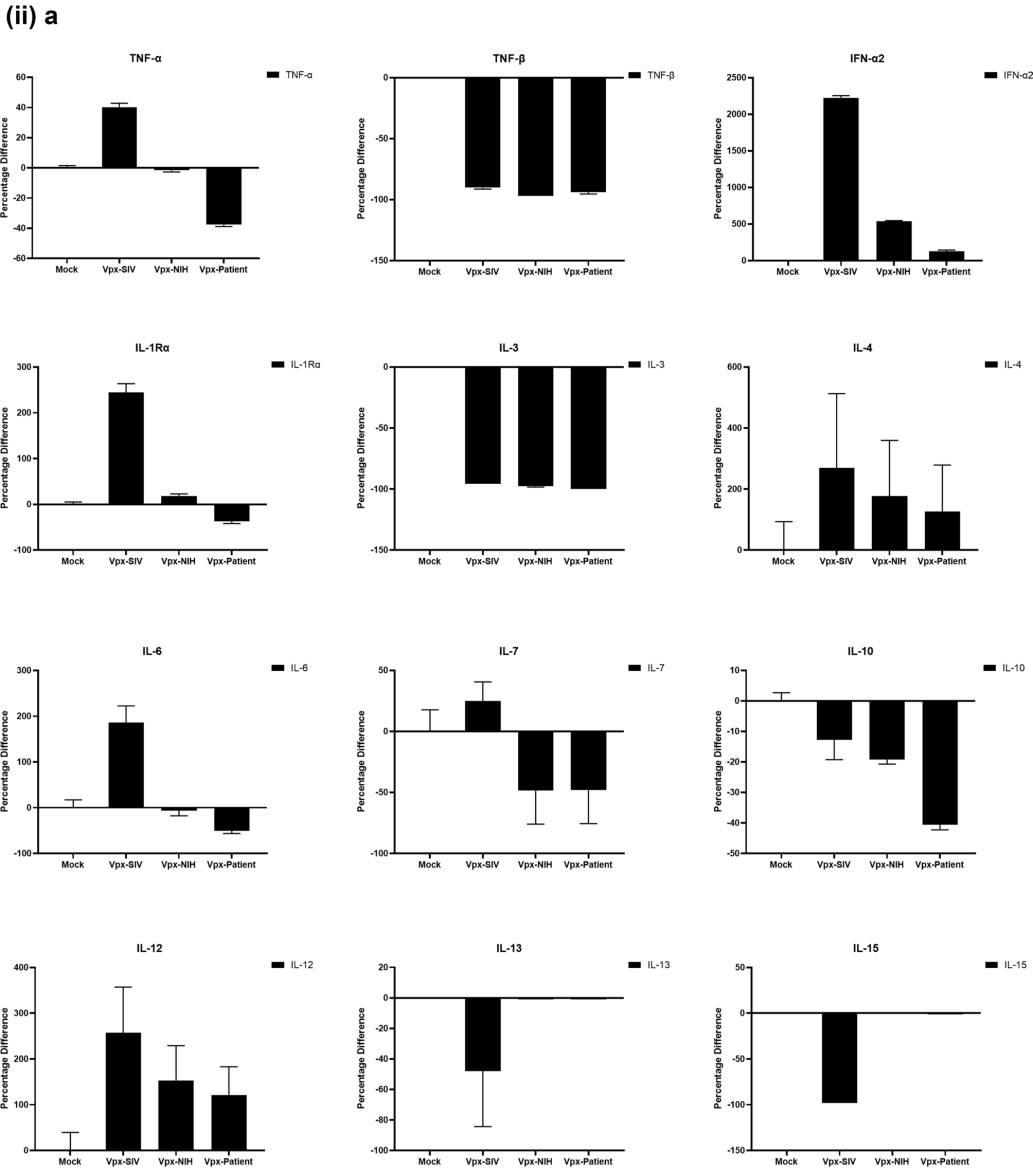

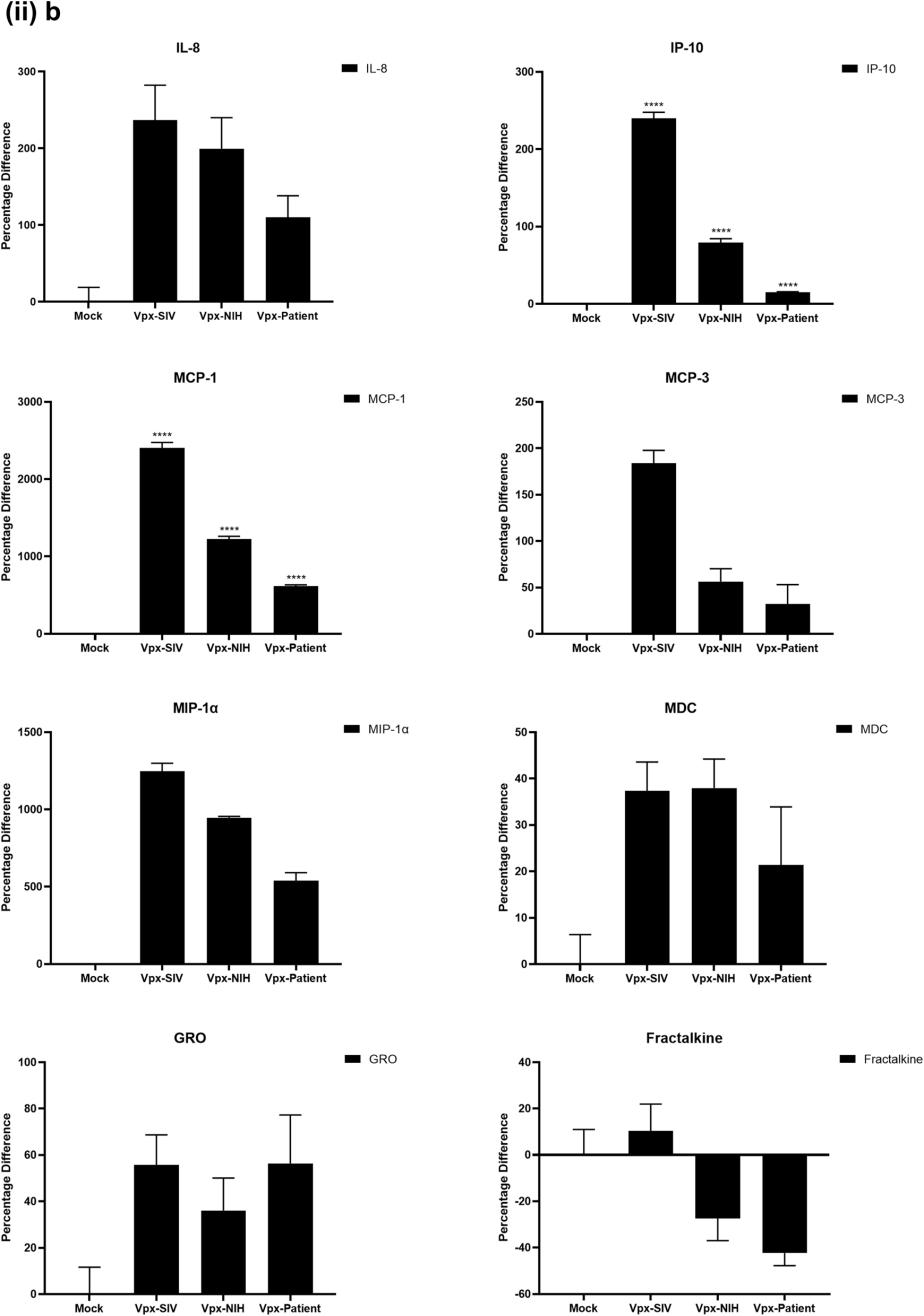

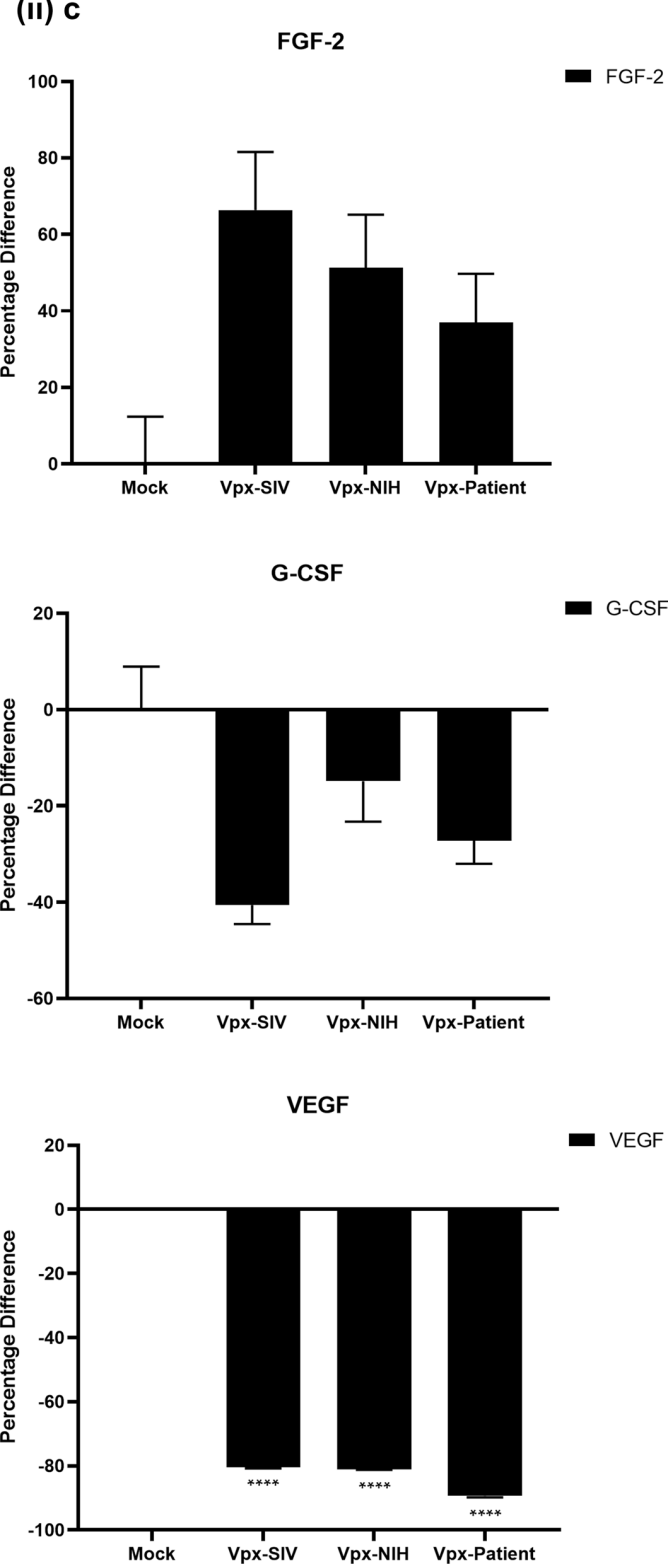
Multiplex cytokine array analysis of supernatants of U937 cells following transfection with pSIVmac-Vpx, pHIV-2-Vpx (NIH), pHIV-2-Vpx (Patient) and mock plasmid. Supernatants were collected after 24 and subjected to analyte analysis by Multiplex cytokine array analysis. (**i**) set represents actual values while (**ii**) represents normalised values against mock and expressed in percentage. Graph (**a**) represents the Cytokines, (**b**) represents Chemokine and (**c**) represents different levels of growth factors.

To further understand the immune response of SAMHD1 positive THP-1 and SAMHD1 negative U937 (Fig. 5) cells following transfection with Vpx gene, we cultured both cell lines for 24 h, collected their supernatants, and analyzed for secreted cytokines, chemokines, growth factors and soluble ligands using Multiplex array (Fig. 4 (i)a–c). Using 24 h as a reference point for secreted cytokines and chemokines, we found that in THP-1 cells, SIVmac were considerably upregulating pro-inflammatory cytokines, including TNF-α, IL-1β, IL-6, IL-12, IL-8, MCP-1, MIP-1α, GRO, Fractalkine, MCP-3, IL-15, IFN-α2, and FGF (Fig. 4 (ii)a–c). However, these pro-inflammatory cytokines were comparatively reduced in HIV-2 transfectants (pHIV-2-Patient as well as pHIV-2-NIH). The most striking immune response was of TNF-α which was considerably suppressed in SIVmac-Vpx and NIH-HIV-2-Vpx, but dramatically upregulated in Saudi patient HIV-2-Vpx transfectants. The effects of Vpx transfection on the levels of MDC, IL-1RA, G-CSF and VEGF were not significant (Fig. 4). Interestingly, IL-10 levels were up in SIVmac-Vpx transfectants compared to HIV-2-Vpx transfected THP-1 cells. These results suggest that Vpx derived from HIV-2 may be responsible for suppressing pro-inflammatory cytokines, thereby promoting an anti-inflammatory milieu for latency in the infected cells. To identify whether this effect was Vpx- independent, we measured cytokines, chemokines and growth factors in the supernatant of the SAMHD1 negative U937 cells (Fig. 5). To our surprise, TNF-α expression was highly expressed in HIV-2 (NIH) transfectants while its expression remained low in-HIV-2 patient construct transfected in U937 cells. VEGF and IL-1RA were considerably reduced in HIV-2-Vpx transfectants compared to SIVmac. These results suggest that Vpx affects cytokine/chemokine response in macrophages in a way that suits the quiet, non-inflammatory survival of HIV-2. In addition, SIVmac mediated immune response was very distinct from HIV-2-Vpx mediated triggering of cytokine/chemokine responses in macrophages (Fig. 5).

## Discussion

In this study, we cloned Vpx gene from an HIV-2 infected Saudi patient and characterized its sequence revealing gene diversity and variability. We also report that Vpx led to reduced levels of intracellular SAMHD1 in permissive cells. When the supernatants of the transfected permissive cell lines were examined for cytokines, chemokines and growth factors using multiplex array, Vpx expression seemed to be suppressive of pro-inflammatory response and skewed the immune response towards an anti-inflammatory response. Our results suggest that Vpx can help clear intracellular restriction factor and suppress of cytokine storm, both favoring latency.

SAMHD1 is highly expressed in primary B-lymphocytes, CD4+ T-lymphocytes, and CD14+ monocytes^27^, monocyte-derived macrophages and DCs^3,4,24,28–32^. SAMHD1 was first cloned from human DCs as an IFN-γ-inducible gene^33,34^, acting as a negative regulator of the IFN response^33^. It was shown that the cellular exonuclease TREX1 bound and degraded excess cytosolic HIV-1 DNA that could induce type I IFN production, triggering innate immune responses against viral infection^35^. Thus, HIV-1 can potentially evade innate immunity in the infected cells through TREX1-mediated viral DNA degradation.

Vpx, exclusive to HIV-2 and SIVmac, seems to have originated via Vpr gene duplication^36^. In macrophages and DCs, Vpx-deficient virus is blocked before reverse transcription sets in. This can be circumvented by providing cells Vpx via virus-like particles^22,37^. It appears that SAMHD1 is the target for proteolysis engineered by Vpx^3,4,24^. This is evident when gene silencing of SAMHD1 in macrophages/DCs raises their susceptibility to infection by Vpx-deficient virus^3,24^. Vpx also enhances HIV-1 infection of DC/CD4+ T-cells^17,20,28,29,38,39^. In susceptible cells, SAMHD1 is proteasomally degraded by Vpx via the ubiquitin ligase complex^3,24^. Thus, SAMHD1 deficiency enhances HIV-1 infection^3,4,24,28^. Vpx recruits CUL4A-DDB1-DCAF1 (DDB1 and CUL4 Associated Factor 1) E3 ubiquitin ligase^23^, binds to SAMHD1, and facilitates its degradation. For this degradation to take place, SAMHD1 should be localized in the nucleus^22,40,41^.

Overexpression of SAMHD1 in PMA-treated U937 cells results in depletion of dNTP levels^42^, which restricts the replication of retroviruses and several DNA viruses^3,24,42,43^. An additional mechanism of SAMHD1-mediated retroviral restriction has^44^ also been described that is dependent on phosphorylation^44^. SAMHD1 acts as an ssDNA binding protein that degrades ssDNA and RNA via a metal-dependent 3′–5′ exonuclease activity in vitro^14,15,45^. SAMHD1 utilizes its nucleic acid binding ability to cleave HIV-1 genomic RNA in a phosphorylation-dependent manner^16^. SAMHD1 also restricts retroviruses though degradation of HIV-1 RNA in human monocyte-derived macrophages, monocytes, and CD4+ T-cells^16,46^.

Toll-like agonists and IFNs can induce SAMHD1 expression^47–49^. Cell lines treated with IFN-γ^49^, and human primary monocytes treated with IFN-α and IFN-γ^50^, show enhanced expression of SAMHD1. While SAMHD1 is highly expressed in MDMs, monocyte-derived DCs, and primary CD4+ T-cells, IFN treatment does not increase SAMHD1 protein levels further. As SAMHD1 limits HIV-1 cDNA synthesis in myeloid cells^42^, degradation of SAMHD1 by Vpx in DCs results in HIV-1 infection, synthesis of viral proteins, their antigen presentation, and subsequent T-cell response^51^. This probably explains why the Vpx gene was lost from the ancestor of HIV-1 during the co-evolution of primate SAMHD1 and lentiviruses^52^.

To further understand the innate immune response associated with SAMHD1-Vpx interaction, we cloned Vpx gene from a HIV-2 infected patient (PBMC) and transfected two macrophage cell lines, THP-1 (SAMHD1+ve) and U937 (SAMHD1−ve), with Vpx containing plasmids sourced from SIVmac, patient HIV-2, and a reference NIH Vpx. We examined the effect of Vpx transfection of the expression and sustenance of SAMHD1 in the macrophage cell lines, and their subsequent immune response following Vpx transfection via cytokine/chemokine/growth factor multiplex array analysis. Cloning, sequencing and sequence alignment of the Vpx gene cloned from Saudi patient showed high degree of diversity (> 30%) compared to Vpx from SIVmac, (pSIVmac), pHIV-2-Patient and pHIV-2-NIH. We then carried out transfection of SAMHD1 positive THP-1 as well as SAMHD1 negative U937 cell lines with Vpx plasmid sourced from SIVmac, NIH strain and Saudi patient. Western blot using total extract of various transfectants revealed that THP-1 cells expressed SAMHD1 consistently in good amounts; this was considerably lowered following Vpx transfection. The levels of SAMHD1 expression varied among SIVmac, Saudi patient and NIH reference Vpx, corresponding to their likely proteosomal degradation by Vpx. The most dramatic effect was evident in the Saudi patient Vpx group at 24 h and 48 h where poor and almost no SAMHD1 expression was detectable, respectively, by western blot. SAMHD1 was not detected in all the samples originating from U937 cells. We wanted to confirm these results derived from total extract in a native context. Thus, FACS was used to analyze THP-1 cell line, transfected with Vpx genes derived from SIVmac, Saudi patient HIV-2 and the NIH reference HIV-2. Vpx expression led to reduced level of detection of SAMHD1 in permeabilized THP-1 cells; these effects were consistent in patient HIV-2 Vpx transfectants but quite dramatic in the reference NIH strain.

To examine the immune response of SAMHD1 positive THP-1 and SAMHD1 negative U937 cells following transfection with Vpx gene, we cultured both cell lines and their supernatants were measured for secreted cytokines, chemokines, growth factors and soluble ligands using Multiplex array. At 48 h time point, we found that in THP-1 cells, SIVmac-Vpx were considerably upregulating pro-inflammatory cytokines including TNF-α, IL-1α, IL-6, IL-12, IL-8, MCP-1, MIP-1 α, GRO, Fractalkine, MCP-3, IL-15, IFN-α 2, and FGF. These cytokines, chemokines and growth factors were comparatively reduced in HIV-2-Vpx transfectants (NIH as well as Saudi patient). Interestingly, TNF- α was considerably suppressed in SIVmac-Vpx and NIH-HIV-2-Vpx, but dramatically upregulated in Saudi patient HIV-2-Vpx transfectants. The effects of Vpx transfectants on the levels of MDC, IL-1RA, G-CSF and VEGF were not differential. Curiously, IL-10 levels were upregulated in SIVmac-Vpx transfectants compared to HIV-2-Vpx transfected THP-1 cells. Thus, Vpx derived from HIV-2 may be responsible for suppressing cytokine storm. In other words, Vpx may promote an anti-inflammatory milieu for latency in the infected cells, an immunological mechanism dissimilar to HIV-1. It appears that restriction of SAMHD1 and suppression of pro-inflammatory immune response are taking place in parallel; SAMHD1 non-expressing U937 cells were able to mount an immune response similar to THP-1 cells when challenged with Vpx. Further experiments are required to validate these proposed mechanisms. In the case of U937 cells, the results mirrored the observations of THP-1 cells with few exceptions. For instance, TNF- α was not suppressed in NIH-HIV-2-Vpx transfectants while it was low in Saudi patient HIV-2-Vpx U937 cells. VEGF and IL-1RA were considerably reduced in HIV-2-Vpx transfectants compared to SIVmac-Vpx.

## Conclusion

We show that Vpx cloned from HIV-2 infected PBMCs from Saudi patient have considerable sequence divergence. It can degrade SAMHD1 inside the cells, and mount an anti-inflammatory response in monocyte-like macrophage cell lines. Vpx seems to affect cytokine/chemokine response in macrophages in a way that suits the quiet, non-inflammatory survival of HIV-2. Our results highlights the importance of Vpx-SAMHD1 interaction in HIV-2-mediated immunosuppressive environment that is conducive to maintenance of viral latency.

## Methods

### Blood collection and sample processing

HIV-2 infected patient from the King Faisal Specialist Hospital & Research Center (KFSH&RC), Riyadh (n = 1) and one negative healthy control person. Patient specimens were collected and analyzed following written consent by patients. The parient (45 year old) was under highly active antiretroviral therapy (HAART). HIV blood samples were collected in green top blood tubes (sodium heparin anticoagulant) to separate the blood component by ficoll (GE Healthcare) into peripheral blood mononuclear cells (PBMCs), red blood cells (RBCs) and plasma.

### Tissue culture

THP-1 (ATCC Cat no. TIB-202) and U937 (ATCC Cat no. CRL-1593.2) were procured from American Type Culture Collection (ATCC). THP-1 and U937 cells were maintained in Roswell Park Memorial Institute medium (RPMI) medium with l-glutamine (Sigma) supplemented with 10% v/v fetal bovine serum from (Gibco cat. # 26140079) and 1% v/v Penicillin–Streptomycin (Sigma; # P4333). Cells were grown at 37 °C with 5% v/v CO_2_ until 80% confluence.

### Plasmids constructs

Vpx gene from HIV-2 NIH viral stock (pHIV-2-NIH) as well as Saudi HIV-2 patient plasma (pHIV-2-Patient) were amplified, using the following primers: Round (1): Forward Primer: 5′-ATG GAG GAA GGC AAG AAC TG-3′ and Reverse Primer: 5′-TTA GAC TAG ACC TGG AGG GG-3′; and in Round (2) with Restriction enzyme sequence of EcoR-1 within the Forward Primer (Underlined): 5′-AGG ATC CAT GAC AGA CCC CAG-3′ and the Restriction enzyme sequence of BamH-1 within the Reverse Primer (Underlined): 5′-AGA ATT CTT AGA CTA GAC CTG-3′ (gene bank number: AY509260.1), followed by sub-cloning in pcDNA3.1 (+) (Invitrogen). Nested PCR was performed using the above-mentioned primers and cloning sites involved restriction digestion with EcoR-1 and BamH-1 (New England Biolabs). The pSIVmac (Vpx) construct was kindly provided by Dr. Nathaniel R Landau (NYU Health Sciences, USA).

### Transfection

Lipofectamin LTX (Thermofisher cat no. 15338100) was used for the transfection of cell lines using recombinant DNA. 5 × 105 THP-1 and U937 cells were added in 1.5 ml RPMI growth medium in a 6-well plate. In each well, a mixture of recombinant DNA with 0.5 ml of Opti-MEM medium and 2.5 µl plus reagent (Lipofectamin Kit) was added. Next, 11 µl of lipofectamin was added to the mixture and incubated for 25 min; the mixture was then added to the cells, mixed gently and incubated at 37 °C under 5% v/v CO_2_ for 24 h or 48 h.

### Western blot

THP-1 and U937 cells were transfected with pSIVmac, pHIV-2-Patient, pHIV-2-NIH and mock plasmid, and then incubated for 24 h. Proteins were extracted by using cell lysis buffer (Radio immunoprecipitation assay (RIPA)) (Sigma-Aldrich cat no. R0278) containing protease cocktail inhibitors (Sigma-Aldrich cat no. P8340). After measuring the concentration at 595 wave lengths, 50 μg of proteins and pre-stained protein ladder (Bio rad cat no. 1610377) were separated by electrophoresis and transferred to the PVDF membrane. Anti-SAMHD1 (1:500) (Abcam cat no. ab83982), anti-Vpx (1:500) (Mybiosource cat no. P06939) and anti-β-actin (1:1000) (Cell Signaling; cat no. 8H10D10) antibodies were used to probe the western blot.

### Flow cytometry

THP-1 cells transfected with pSIVmac, pHIV-2-Patient, pHIV-2-NIH or mock plasmid were used for flow cytometry. Transfected cells were fixed with 3.7% v/v formaldehyde solution for 15 min and washed with washing buffer [phosphate buffer saline (PBS) + 2% v/v fetal bovine serum (FBS)] three times, then permeabilized with 0.5% v/v Triton X 100 for 5 min, and washed. Primary antibodies (Anti-SAMHD1 and Anti-Vpx from (NIH AIDS Reagent Program cat no. 2710)) (both 1:1000) were added in separate tubes for overnight at 4 °C. Next day, anti-mouse FITC (1:1000) were added as secondary antibody in each tube and incubated for 1 h. The cells were then examined by flow cytometry (BD LSR II). To ensure consistency between analyses, instrument quality control and consistent target fluorescence intensities using control beads were used. Observe cells falling in the monocyte gate of the CD14/CD16 plot. The recording threshold was 5000 events for the classical monocyte gate that were recorded. We then caluclated the gated frequency for the percentage of monocyte and the degree of expression.

### Multiplex cytokine array analysis

Supernatant from THP-1 and U937 cells that transfected with pSIVmac, pHIV-2-Patient, pHIV-2-NIH and mock plasmid, were collected after 24 h, for measuring secreted cytokines, chemokines, and additional immune regulators. The analytes were measured using Milliplex Array kit using Luminex (Merck UK) following the previously published protocol^53^.

### Ethics approval and consent to participate

The study was conducted according to the guidelines of the Declaration of Helsinki. This study was approved by Research Advisory Council (RAC) of King Faisal Specialist Hospital and Research Centre, RAC # 2130003, Informed consent was obtained from all subjects involved in the study.

## Supplementary Information

Supplementary Legends.

Supplementary Figures.

## Data Availability

All data generated or analyzed during this study are included in this published article (and its supplementary information files).
