# Supplementary material for: HIV-2 Vpx neutralizes host restriction factor SAMHD1 to promote viral pathogenesis
Source: Sci Rep. 2021 Oct 25;11:20984. doi: 10.1038/s41598-021-00415-2 (PMC8545964; doi:10.1038/s41598-021-00415-2)
Supplement: Supplementary file 1 — Supplementary Legends. [file 41598_2021_415_MOESM1_ESM.docx]

Article

**HIV-2 Vpx neutralizes host restriction factor SAMHD1 to promote viral pathogenesis**

Ahlam Mohamed^1^, Talal Bakir^2^, Huda Al-Hawel^1^, Ibtihaj Al-Sharif ^1^, Razan Bakheet^1^, Lubna Kouser^3^, Valarmathy Murugaiah^4^, Maha Al-Mozaini^1,2^*

^1^ Immunocompromised Host Research Section, Department of Infection and Immunity, King Faisal Specialist Hospital and Research Centre, Riyadh, Saudi Arabia

^2^ Department of Clinical Laboratories Sciences, College of Applied Medical Sciences, King Saud University, Riyadh, Saudi Arabia.

^3^ Imperial College London, United Kingdom.

^4^ Biosciences, College of Health, Medicine and Life Sciences, Brunel University London.

^*^ Corresponding author: Maha A Al-Mozaini

Immunocompromised Host Research Section

Department of Infection and Immunity,

King Faisal Specialist Hospital and Research Centre

PO Box 3354 (MBC-03), Riyadh 11211, Kingdom of Saudi Arabia

E-mail: mmozaini@kfshrc.edu.sa

**Supplementary Figure S1.** Amplification product of Vpx from patient’s sample and the Sequence analysis of different cloned Vpx genes) PCR amplicon product for Vpr and Vpx genes from HIV-1 and HIV-2 PBMCs patient samples. Vpr amplicon product can be seen present in both HIV-1 and HIV-2 samples, thus, used as control; however, Vpx gene is amplified only in HIV-2, to confirm the viral type.

**Supplementary Figure S2.** Western blot to probe SAMHD1 expression with and without Vpx THP-1 Cell line transfected with pSIVmac-Vpx, pHIV-2-Patient, pHIV-2-NIH and mock plasmid were incubated for 24 and the western blot was probed using anti-SAMHD1 and anti-Vpx monoclonal antibodies; anti- β -actin was used as a housekeeping positive control.

**Supplementary Figure S3.** Western blot to probe SAMHD1 expression with and without Vpx U937 Cell line transfected with pSIVmac-Vpx, pHIV-2-Patient, pHIV-2-NIH and mock plasmid were incubated for 24 and the western blot was probed using anti-SAMHD1 and anti-Vpx monoclonal antibodies; anti- β -actin was used as a housekeeping positive control.

**Supplementary Figure S4.** Western blot for different PBMCs sample from HIV-1, HIV-2 and healthy negative control donor cells. Anti-SAMHD1, anti-Vpx and anti-Beta actin were used. As shown Vpx was express in HIV-2 PBMCs cells protein. SAMHD1 in HIV-2 has low express compared to HIV-1 and donor cells protein. β-actin was used as housekeeping gene control

**Supplementary Figure S5.** Western Blot analysis for SAMHD1 expression with and without Vpx in THP-1 and U937 Cell lines transfected with transfected with pSIVmac-Vpx , pHIV-2-Patient, pHIV-2-NIH and mock plasmid . PBMCs sample from HIV-1, HIV-2 and healthy negative control donor cells. Anti-SAMHD1, anti-Vpx and anti- β actin were used. β-actin was used as housekeeping gene control.
