# Supplementary material for: HIV-2 Vpx neutralizes host restriction factor SAMHD1 to promote viral pathogenesis
Source: Sci Rep. 2021 Oct 25;11:20984. doi: 10.1038/s41598-021-00415-2 (PMC8545964; doi:10.1038/s41598-021-00415-2)

Supplementary Figure S1

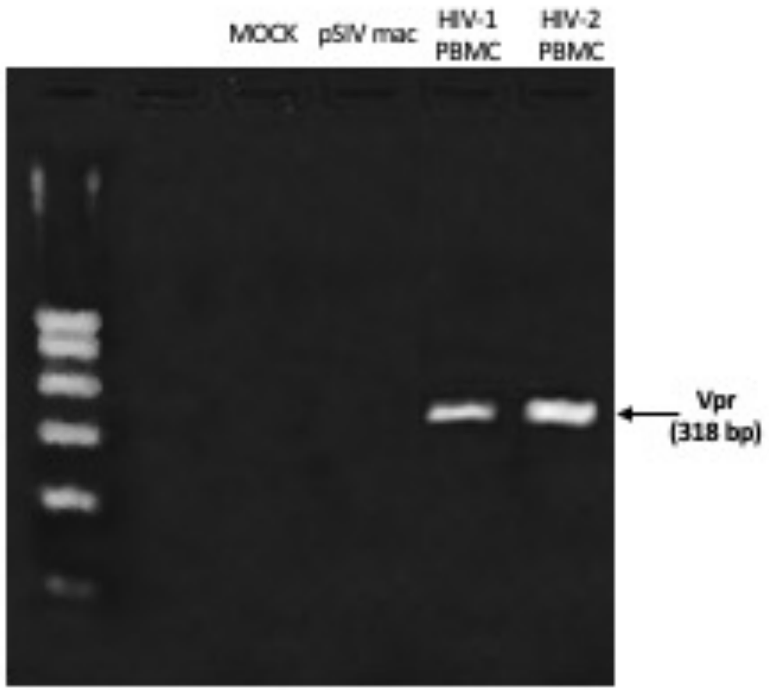

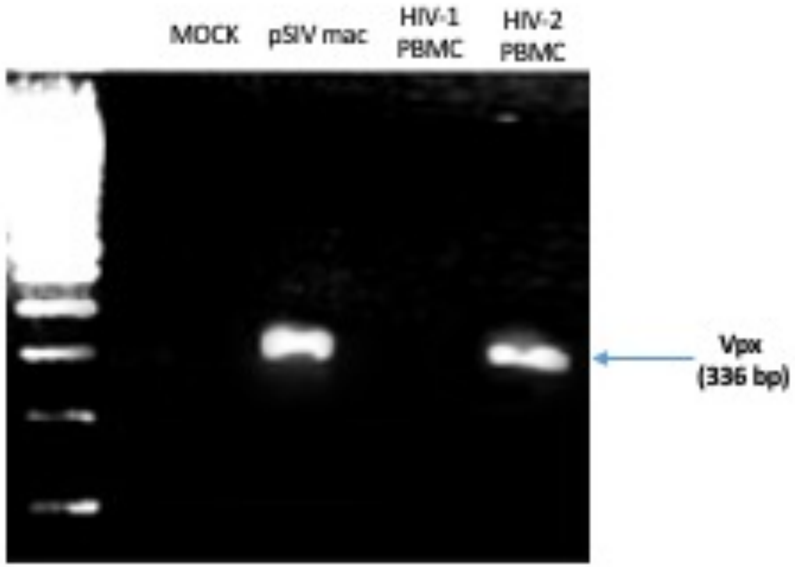

Supplementary Figure S2

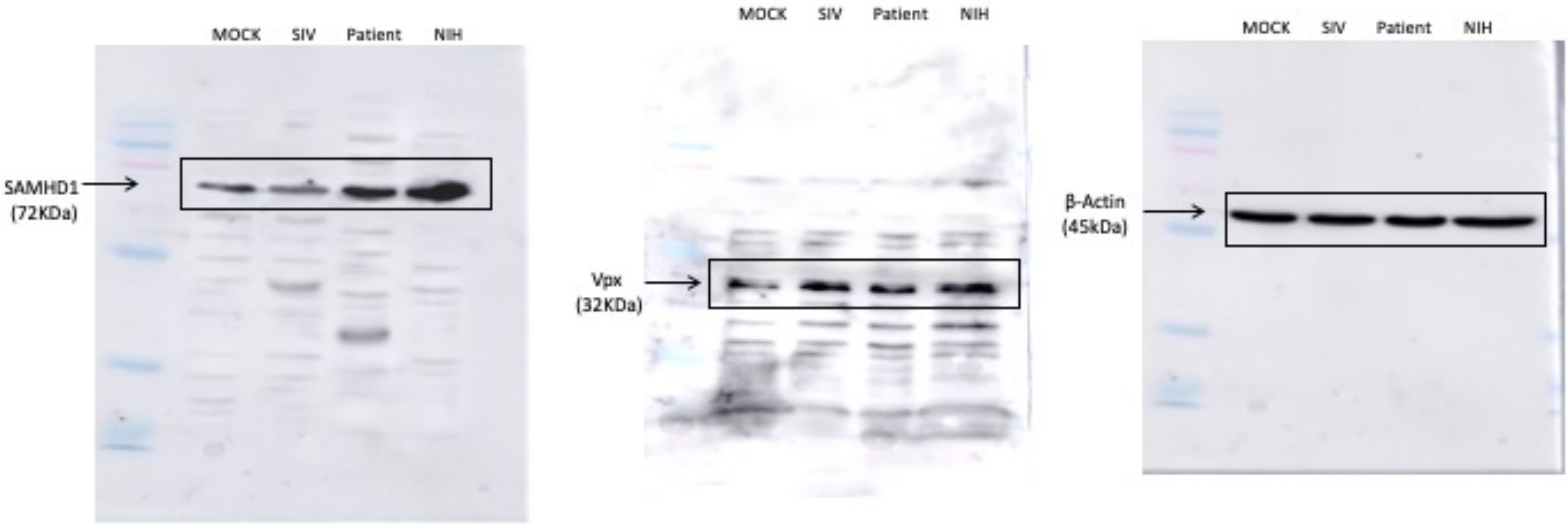

Supplementary Figure S3

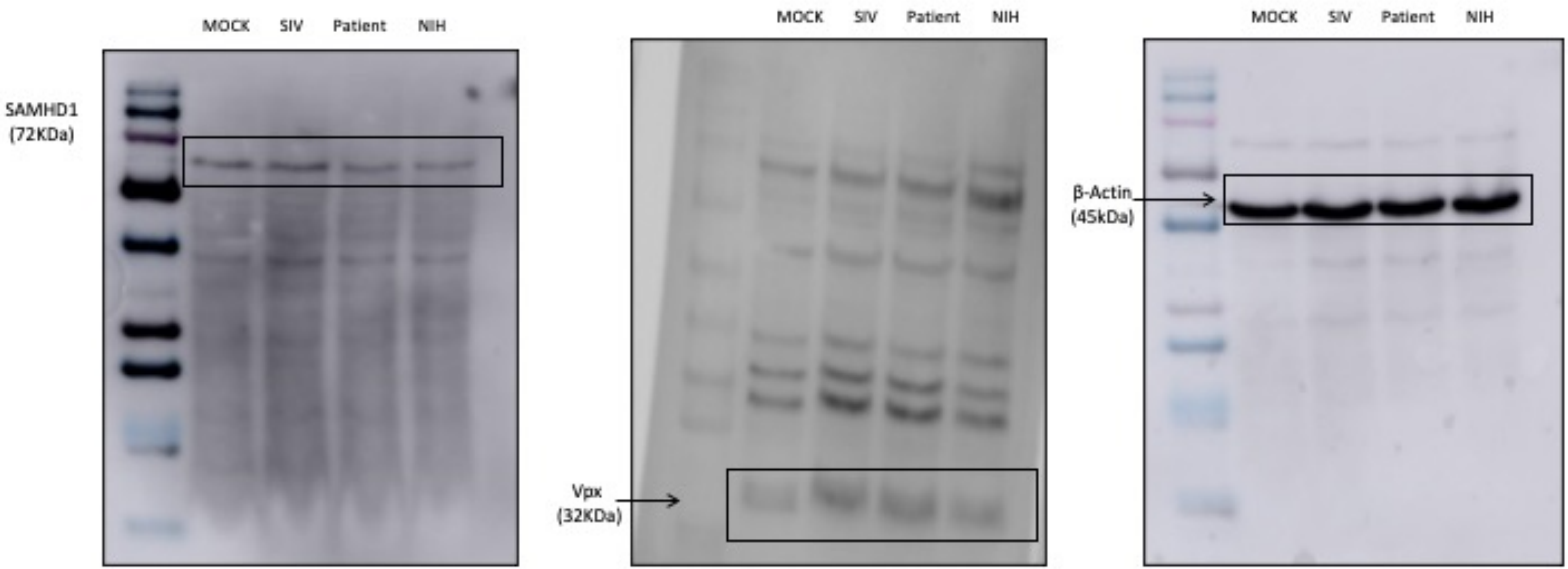

Supplementary Figure S4

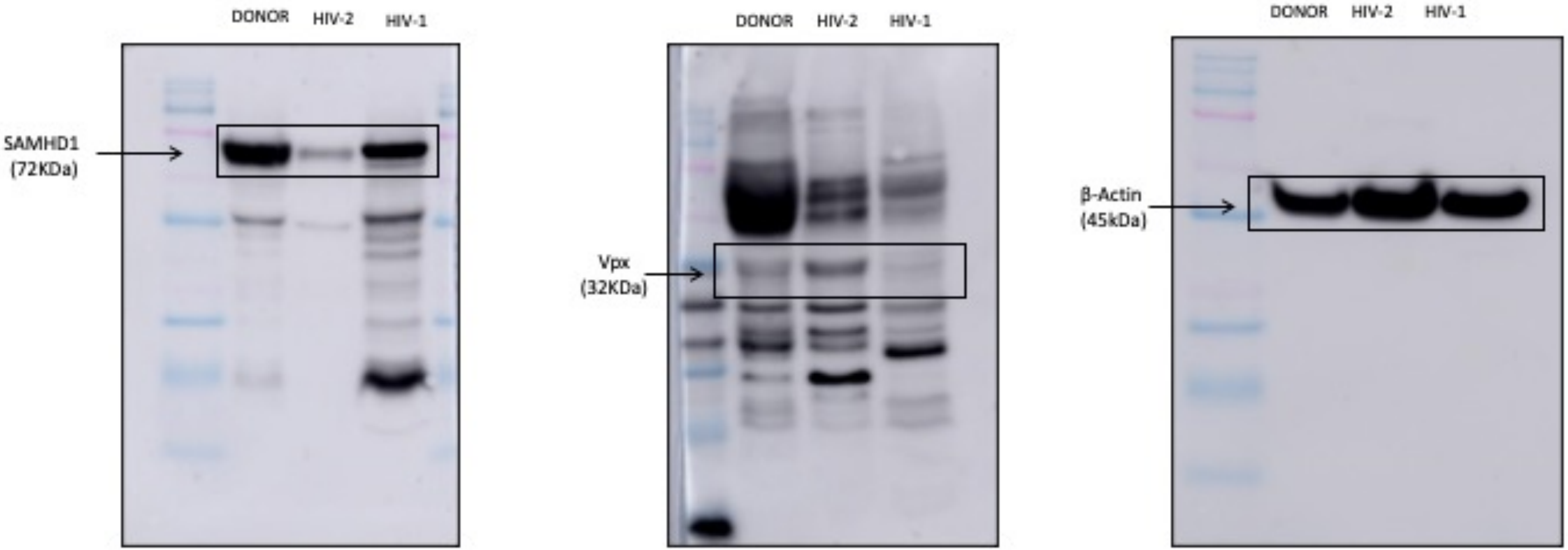

Supplementary Figure S5

THP-1				
	**MOCK**	**pSIVmac**	**pHIV-2-Patient**	**pHIV-2-NIH**
SAMHD-1	120710.3	107163.3	184587.3	173963.3
Fold Increase		-0.1	0.5	0.4
VPX	8522.33	25352.33	23356.33	47332.33
Fold Increase		2.0	1.7	4.6
Actin	160941.0	169303.0	157596.0	173348.0
Fold Increase		0.1	0.0	0.1

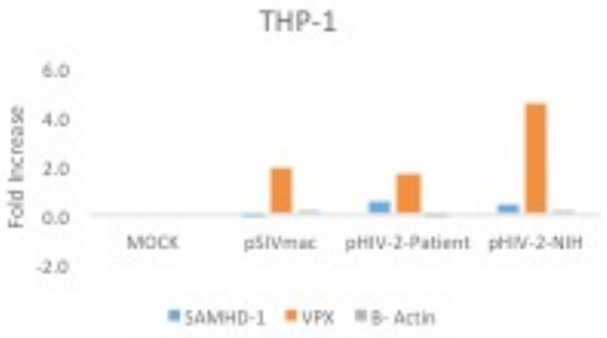

U937				
	**MOCK**	**pSIVmac**	**pHIV-2-Patient**	**pHIV-2-NIH**
SAMHD-1	10252.3	15927.3	5893.3	4712.3
Fold Increase		0.6	-0.4	-0.5
VPX	12807.00	30923.00	31194.00	22562.00
Fold Increase		1.4	1.4	0.8
Actin	132895.78	134957.44	134827.78	134915.78
Fold Increase		0.0	0.0	0.0

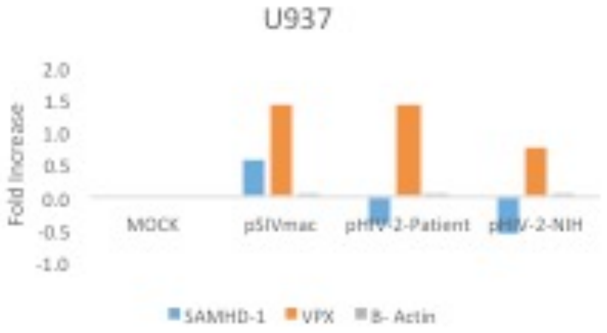

PBMCS			
	**-ev control**	**HIV2**	**HIV1**
SAMHD-1	164068.0	17457.3	161186.7
Fold Increase		-0.9	0.0
VPX	15565.2	113512.6	-18073.4
Fold Incre m		6.3	-2.2
Actin	158477.3	195878.7	192565.3
Fold Increase		0.2	0.2

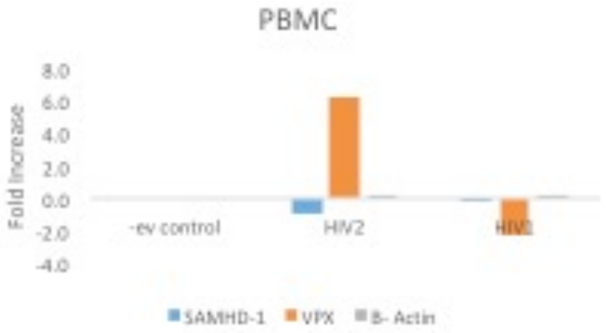

Supplement: Supplementary file 2 — Supplementary Figures. [file 41598_2021_415_MOESM2_ESM.pdf]
